# Supplementary material for: Positively Selected Effector Genes and Their Contribution to Virulence in the Smut Fungus Sporisorium reilianum
Source: Genome Biol Evol. 2018 Jan 30;10(2):629–45. doi: 10.1093/gbe/evy023 (PMC5811872; doi:10.1093/gbe/evy023)
Supplement: Supplementary Tables and Figures [file evy023_supp.zip › SupplementaryTableS4.docx]

**Supplementary table S4:** List of strains of *S. reilianum* f. sp. *zeae* used in the present study

| Strain name | Relevant genotype | Resistance^1^ | Reference |
| --- | --- | --- | --- |
| JS161 | *a1 mfa2.1 bW1 bE2* | P | Schirawski *et al*., 2010 |
| JS161ΔSr10529 | *a1 mfa2.1 bW1 bE2, sr10529::gen* | P, G | This work |
| JS161ΔSr12968 | *a1 mfa2.1 bW1 bE2, sr12968::gen* | P ,G | This work |
| JS161ΔSr14944 | *a1 mfa2.1 bW1 bE2, sr14944::gen* | P, G | This work |
| JS161ΔSr10059 | *a1 mfa2.1 bW1 bE2, sr10059::gen* | P, G | This work |
| JS161ΔSr10182 | *a1 mfa2.1 bW1 bE2, sr10182::gen* | P, G | This work |
| JS161ΔSr14558 | *a1 mfa2.1 bW1 bE2, sr14558::gen* | P, G | This work |
| JS161ΔSr14347 | *a1 mfa2.1 bW1 bE2, sr14347::gen* | P, G | This work |
| JS161ΔSr12897 | *a1 mfa2.1 bW1 bE2, sr12897::gen* | P, G | This work |
| JS161ΔSr12084 | *a1 mfa2.1 bW1 bE2, sr12084::gen* | P, G | This work |

^1^ Phleomycin resistance (P), Geneticin (G418) resistance (G)
