# Supplementary figures and images for: Positively Selected Effector Genes and Their Contribution to Virulence in the Smut Fungus Sporisorium reilianum
Source: Genome Biol Evol. 2018 Jan 30;10(2):629–45. doi: 10.1093/gbe/evy023 (PMC5811872; doi:10.1093/gbe/evy023)

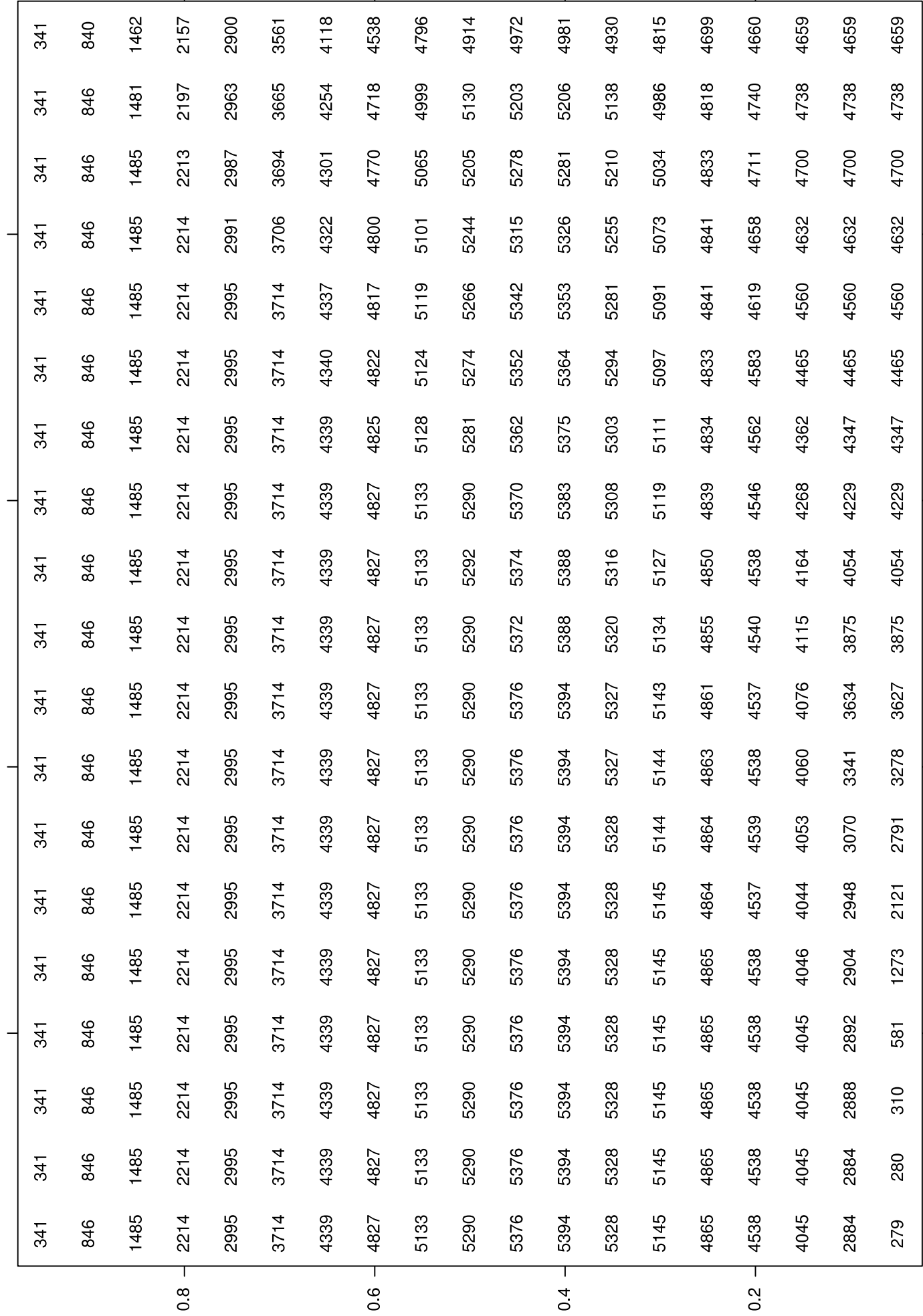

Supplement: Supplementary Tables and Figures [file evy023_supp.zip › SupplementaryFigure1.pdf]

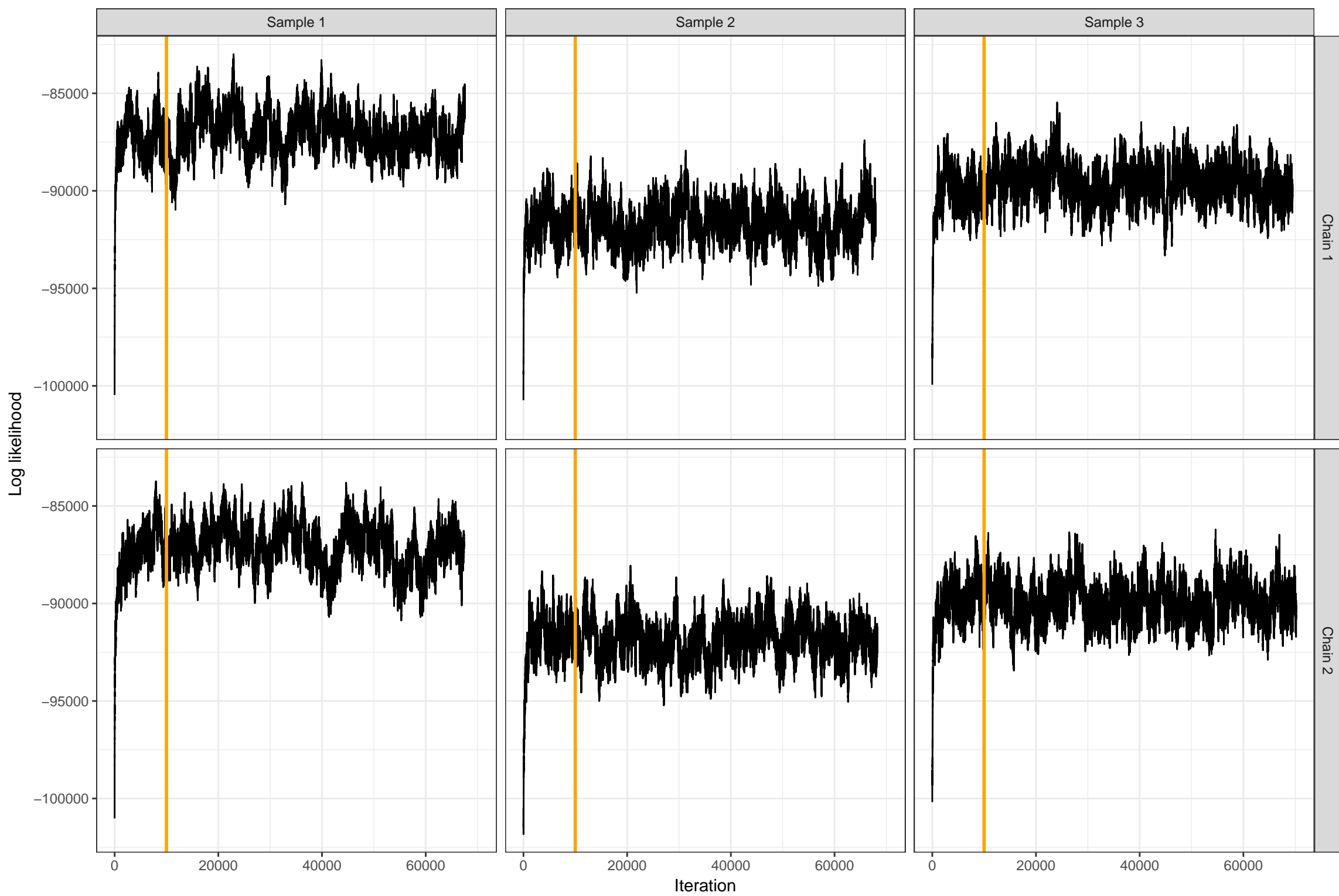

Supplement: Supplementary Tables and Figures [file evy023_supp.zip › SupplementaryFigure2.pdf]
